# Effects of Different Combinations of Sugar and Starch Concentrations on Ruminal Fermentation and Bacterial-Community Composition *in vitro*

**DOI:** 10.3389/fnut.2021.727714

**Published:** 2021-09-03

**Authors:** Jia-nan Dong, Song-ze Li, Xue Chen, Gui-xin Qin, Tao Wang, Zhe Sun, Di Wu, Wei Zhao, Natnael Demelash, Xue-feng Zhang, Yu-guo Zhen

**Affiliations:** ^1^Key Laboratory of Animal Nutrition and Feed Science of Jilin Province, Key Laboratory of Animal Production Product Quality and Security Ministry of Education, JLAU-Borui Dairy Science and Technology R&D Center, College of Animal Science and Technology, Jilin Agricultural University, Changchun, China; ^2^Postdoctoral Scientific Research Workstation, Feed Engineering Technology Research Center of Jilin Province, Changchun Borui Science and Technology Co. Ltd., Changchun, China; ^3^College of Life Science, Jilin Agricultural University, Changchun, China; ^4^Institute of Agricultural Quality Standard and Testing Technology, Jilin Academy of Agricultural Sciences, Changchun, China

**Keywords:** sugar, starch, rumen fermentation parameters, bacterial-community composition, *in vitro*

## Abstract

High levels of starch is known to have positive effects on both energy supply and milk yield but increases the risk of rumen acidosis. The use of sugar as a non-structural carbohydrate could circumvent this risk while maintaining the benefits, but its effects and that of the simultaneous use of both sugar and starch are not as well-understood. This study aimed to evaluate the effects of different combinations of sugar and starch concentrations on ruminal fermentation and bacterial community composition *in vitro* in a 4 ×4 factorial experiment. Sixteen dietary treatments were formulated with 4 levels of sugar (6, 8, 10, and 12% of dietary dry matter), and 4 levels of starch (21, 23, 25, and 27% of dietary dry matter). Samples were taken at 0.5, 1, 3, 6, 12, and 24 h after cultivation to determine the disappearance rate of dry matter, rumen fermentation parameters and bacterial community composition. Butyric acid, gas production, and *Treponema* abundance were significantly influenced by the sugar level. The pH, acetic acid, and propionic acid levels were significantly influenced by starch levels. However, the interactive effect of sugar and starch was only observed on the rate of dry matter disappearance. Furthermore, different combinations of starch and sugar had different effects on volatile fatty acid production rate, gas production rate, and dry matter disappearance rate. The production rate of rumen fermentation parameters in the high sugar group was higher. Additionally, increasing the sugar content in the diet did not change the main phylum composition in the rumen, but significantly increased the relative abundance of Bacteroidetes and Firmicutes phyla, while the relative abundance of Proteobacteria was reduced. At the genus level, the high glucose group showed significantly higher relative abundance of Treponema (*P* < 0.05) and significantly lower relative abundance of Ruminobacter, Ruminococcus, and Streptococcus (*P* < 0.05). In conclusion, different combinations of sugar and starch concentrations have inconsistent effects on rumen fermentation characteristics, suggesting that the starch in diets cannot be simply replaced with sugar; the combined effects of sugar and starch should be considered to improve the feed utilization rate.

## Introduction

Starch and sugar are non-structural carbohydrates (NSCs), and are the main energy source for ruminants. Starch is the primary source of NSC in the diets of lactating dairy cows (1). High concentration NSC diets are fed to ruminants to promote short-chain fatty acid production within the rumen, consequently increasing the energy supply. Sánchez-Duarte et al. (2) conducted a high NSC diet experiment with starch and found that the daily milk yield in the 27%-starch group was more than 3.1 kg/d with less 0.35% fat content than that in the 21%-starch group. Besides, the rate of body weight gain in the high starch groups was over 2 kg/d during the finishing period (3). NSC levels are usually increased by increasing starch levels, which in turn increases the risk of rumen acidosis (4). Sugar is another major component of NSC, and its supplementation may also be used as a method to increase NSC levels. Sugars comprise monosaccharides (glucose, fructose, and galactose) and disaccharides (sucrose, maltose, and lactose), which are water-soluble carbohydrates that can be fermented quickly and easily in the rumen (5). The Cornell Net Carbohydrate and Protein System shows that the degradation rate of sugar (including sugar beet and molasses) is 0.40, and that of starch is 0.10–0.35, indicating that the degradation rate of sugar is faster than that of starch (6). Therefore, it is predicted that sugar can quickly produce volatile fatty acids (VFAs) that reduce ruminal pH and increase the risk of rumen acidosis (5). However, most studies have shown that increasing the sugar level in the diet cannot significantly reduce the pH of the rumen fluid (7), while it can significantly increase the concentration of butyric acid in the rumen (8–10). Münnich (11) conducted an experiment with feeding sugar beet pulp and noted that a high-sugar diet increased the yield and concentration of milk fat without affecting the fat composition in milk.

There are few studies on the effect of the combination of sugar and starch, and their interaction in diets. Therefore, the aim of the present study was to assess the effect of different combinations of sugar and starch on ruminal fermentation *in vitro*, and examined whether the different combinations can change bacterial community composition.

## Materials and Methods

### Animals and Experimental Design

Ruminal fluid for this experiment was obtained from three male small-tailed Han sheep (body weight 45 ± 2 kg; age 12 ± 1 months) fitted with a ruminal fistula. Animals were kept in individual rearing cages and were given free access to clean water. All experimental procedures were according to the Guidelines for the Care and Use of Experimental Animals of Jilin Agricultural University.

The experiments were conducted *in vitro* using a 4 ×4 full factorial experimental design comprising a total of 16 groups of rations (Supplemental Table 1). Three replicates were examined for each group. Ingredients and chemical composition of the experimental diets (% of dry matter) are as shown in Supplemental Table 2.

### Collection of Rumen Fluid, Measurement of Dry Matter (DM) Digestibility, and *in vitro* Experiment

Before feeding in the morning, a polyvinyl chloride tube was used to collect rumen fluid from different points in the rumen of the sheep *via* the ruminal cannula. The rumen fluid was filled in thermos bottles (pre-heated to 39°C, filled with CO_2_ gas), the bottle cap was covered immediately, and the bottles were returned to the laboratory quickly. A 5 L beaker was preheated in a 39°C water bath, and the collected rumen fluid was filtered into the beaker through four layers of cheesecloth. CO_2_ was passed to ensure an anaerobic environment, and the rumen fluid filtrates of the three sheep were mixed well to obtain the rumen inoculum. *In vitro* digestibility of samples was measured using the Ankom DaisyII Incubator system (Ankom Technology Corp., Fairport, NY). The weight of each F58 filter bag was measured (pore size = 10 μm; Ankom Technology Corp) and recorded as W_1_. Then, 1 g of the sample (W_2_) was weighed and placed directly into the filter bag. The bags were sealed using heat (model #AIE-200, American International Electric, City of Industry, CA) and were placed in the DaisyII Incubator digestion jar. Three sealed blank bags were included in each digestion jar for correction factor (C1). The incubation jars were pre-heated to 39°C before beginning the experiment. The buffer was prepared anaerobically as described by Menke and Steingass (12), and each incubation jar was filled with 1,200 mL of the buffer and 400 mL of rumen inoculum. Then, the digestion jar was filled with CO_2_ gas for 30 s while ensuring that the lid was secure. Then, triplicate samples from each group were collected after incubation for 0.5, 1, 3, 6, 12, or 24 h, followed by rinsing in fiber bags with clean water until the water was clear and drying in a convection oven at 105°C to constant weight. The *in vitro* weight post the experiment was recorded as W_3_. Thereafter, the *In Vitro* True Digestibility (IVTD) was determined based on the following equation.

$$\begin{array}{l} \%IVTD(DMbasis)=\frac{100\;-\;[W3\;-\;(W1\;\times \;C1)]}{W2\;\times \;DM}\;\times \;100\% \end{array}$$

Where, *W*_1_ = Bag tare weight;

*W*_2_ = Sample weight;

*W*_3_ = Final bag weight after *in vitro* experiment;

*C*_1_ = Blank bag correction (final oven-dried weight/original blank bag weight).

The ANKOM RFS gas production system (Ankom Technology Corp., Fairport, NY) was used for the *in vitro* experiment. Two grams of the different diets (the ingredients and chemical composition of the diets are as presented in Table 1) was placed in the bottles and filled with 40 mL ruminal inoculum and 80 mL buffer, followed by continuous filling of the bottle with CO_2_ for 30 s. All fermenters were then incubated at 39°C in an air bath with shaking at 80 rpm. Simultaneously, the GPM software was used to monitor and record gas production, which was determined based on the following equation.

$$\begin{array}{lll} V_{\text{X}} & = & V_{\text{j}}\times P_{\text{psi}}\times 0.068004084\\ {\text{V}}_{\text{x}} & = & \text{gas produced in mL};\\ {\text{V}}_{\text{j}} & = & \text{the head}-\text{space volume in the glass bottle mL};\\ {\text{P}}_{\text{psi}} & = & \text{psi}. \end{array}$$

**Table 1:** Comparison of DM digestibility (g/kg), pH, and gas production (mL) in each group.

**Item**	**Treatments**																**SEM**	***P*** **-value**		
	**St1 × su1**	**St2 × su1**	**St3 × su1**	**St4 × su1**	**St1 × su2**	**St2 × su2**	**St3 × su2**	**St4 × su2**	**St1 × su3**	**St2 × su3**	**St3 × su3**	**St4 × su3**	**St1 × su4**	**St2 × su4**	**St3 × su4**	**St4 × su4**		**Starch**	**Sugar**	**St × su**
**Dry matter digestibility (g/kg)**																				
0.5	122.9	119.1	111.6	150.2	179.2	112.4	172.8	186.0	160.1	190.8	114.6	175.2	192.9	225.5	200.6	122.6	5.3	<0.01	<0.01	<0.01
1	124.5	124.4	125.6	152.5	192.9	132.9	173.8	188.1	176.6	204.3	154.1	204.6	207.6	226.4	209.9	131.5	5.2	0.26	<0.01	<0.01
3	138.3	124.2	132.5	164.9	217.3	161.5	180.1	193.0	179.3	232.0	175.9	204.7	209.4	227.6	248.0	183.3	5.2	0.90	<0.01	<0.01
6	177.4	148.2	158.8	196.9	276.2	175.1	218.6	242.5	208.0	279.8	213.7	230.2	243.8	268.3	290.3	212.6	6.3	0.36	<0.01	<0.01
12	240.7	201.8	225.5	267.0	313.7	264.1	276.2	314.2	269.5	337.8	251.5	257.6	352.9	321.0	332.9	263.8	6.4	<0.01	<0.01	<0.01
24	346.1	271.0	309.4	348.1	374.7	306.9	379.3	379.9	360.4	398.0	345.9	340.9	402.0	401.9	387.2	351.1	5.9	0.07	<0.01	<0.01
**pH value**																				
0.5	6.86	6.85	6.83	6.85	6.81	6.80	6.82	6.81	6.72	6.74	6.79	6.76	6.68	6.63	6.67	6.66	0.01	<0.01	0.37	0.35
1	6.77	6.75	6.73	6.80	6.71	6.72	6.71	6.76	6.66	6.66	6.67	6.67	6.57	6.54	6.56	6.56	0.01	<0.01	0.19	0.82
3	6.63	6.63	6.64	6.63	6.59	6.59	6.59	6.59	6.58	6.55	6.55	6.56	6.53	6.53	6.50	6.55	0.01	<0.01	0.70	0.95
6	6.48	6.49	6.58	6.54	6.48	6.43	6.54	6.52	6.36	6.42	6.48	6.47	6.29	6.37	6.43	6.37	0.01	<0.01	<0.01	0.95
12	6.40	6.38	6.38	6.40	6.35	6.36	6.37	6.31	6.28	6.23	6.25	6.29	6.23	6.24	6.23	6.24	0.01	<0.01	0.84	0.41
24	6.04	6.05	6.10	6.10	5.97	6.03	6.06	6.08	5.96	5.99	6.02	6.04	5.91	5.95	6.00	6.00	0.01	<0.01	<0.01	0.96
**Gas production (mL)**																				
0.5	29.78	30.72	29.15	27.11	28.68	31.03	27.74	32.60	28.68	29.31	29.86	30.25	29.39	33.23	32.91	32.76	0.44	0.41	0.10	0.54
1	34.64	35.58	34.79	31.82	32.29	35.73	31.97	36.99	31.97	32.76	34.60	34.17	31.97	38.87	35.26	35.11	0.57	0.37	0.73	0.69
3	54.07	54.39	50.15	49.21	58.01	58.62	55.95	60.34	62.38	62.68	63.97	64.89	69.12	71.00	76.68	76.81	1.31	0.78	<0.01	0.33
6	90.75	88.87	82.44	84.32	97.82	95.61	91.06	94.98	104.54	102.96	102.53	104.08	113.08	115.51	113.51	117.08	1.71	0.36	<0.01	0.93
12	154.69	149.68	140.59	141.69	162.39	155.48	150.78	148.11	164.41	161.90	156.60	157.83	170.68	173.97	174.21	167.08	1.66	<0.01	<0.01	0.57
24	196.07	192.31	181.34	185.57	205.81	195.76	193.56	189.80	207.82	198.88	196.80	200.94	217.94	215.04	206.42	210.80	1.67	<0.01	<0.01	0.90

The fermenters were taken out at 0.5, 1, 3, 6, 12, and 24 h post incubation and the bottle cap was opened instantly. The fermenters were then placed in cold water to stop the fermentation. Next, 5 mL of the samples was collected and stored in a −20°C refrigerator for measuring ammonia nitrogen (NH_3_-N). Additionally, 1 mL of sample was collected and mixed with 200 μL of metaphosphoric acid and stored in a −20°C refrigerator for determining VFAs. The remaining sample was preserved at −80°C for bacterial-community composition analyses.

### Chemical Analysis

A Sanxin MP523-04 pH meter (Shanghai Sanxin Instrumentation, Inc., Shanghai, China) was used to determine the ruminal pH, and a Shimadzu UV-1201 spectrophotometer (Shimadzu, Kyoto, Japan) was used to measure the NH_3_-N concentration, as described by Chaney and Marbach (13). VFAs were analyzed using an Agilent 7890B Gas Chromatograph (Agilent Technologies, Santa Clara, California, USA).

### Model Fitting

Origin (version 2018) software was used to fit sigmoidal curve. The logarithmic model equation fitting degree (*R*^2^) was >0.95, the Gompertz model equation fitting degree (*R*^2^) was >0.90 (gas production, DM digestibility, butyric acid, propionic acid), and the Logistic model equation fitting degree (*R*^2^) was >0.83 (TVFA, acetic acid).

### DNA Extraction and 16S rDNA Gene Sequencing

Total DNA was extracted using the repeated beads and column method (14), and DNA was quantified using a Nanodrop. The quality of DNA was determined using 1.2% agarose gel electrophoresis. The V3–V4 region of the 16S rDNA gene was amplified using the forward primers 338F (5′-ACTCCTACGGGAGGCAG-CA-3′) and reverse primers 806R (5′-GGACTACHVGGGTWTCTAAT-3′). The amplified PCR products were purified using magnetic beads and then used for fluorescence quantification using the Quant iT PicoGreen dsDNA Assay Kit and a microplate reader (BioTek, flx800). The TruSeq Nano DNA LT Library Prep Kit of Illumina company was used to prepare the sequencing library. The purified PCR products were pooled and sequenced using the Illumina MiSeq platform with a MiSeq Reagent Kit v3 at Shanghai Personal Biotechnology Co., Ltd. (Shanghai, China).

### Processing of Sequencing Data

Microbiome bioinformatics were mainly performed with QIIME 2 2019.4 (15). While operational taxonomic unit (OTU) clustering was done using the Vsearch pipeline (v2.13.4), as described previously (16). Briefly, raw sequence data were demultiplexed using the demux plugin followed by primer cutting with cutadapt plugin (17). The sequences were then merged, filtered, and dereplicated using functions of fastq_mergepairs, fastq_filter, and derep_fulllength in Vsearch. All the unique sequences were then clustered at 98% (*via* cluster_size) followed by removing of chimera (*via* uchime_denovo). Finally, the non-chimera sequences were re-clustered at 97% to generate OTU representative sequences and OTU table. The Greengenes database and QIIME2 classify-sklearn algorithm were used: For the representative sequence of each OTU, use the pre-trained Naive Bayes classifier to enter the species annotation. PICRUSt analysis was used to predict functional profiles of rumen bacterial communities. Then according to Kyoto Encyclopedia of Genes and Genomes (KEGG) pathways, predicted genes were summarized. The alpha-diversity metrics [Chao1, Chao (18)], observed species, and Shannon (19, 20) and Simpson (21) indices were estimated using the diversity plugin. Principal coordinates analysis (PCoA) was performed to reveal the differences in the bacterial communities across the four treatments based on Bray–Curtis dissimilarity matrix. The composition of bacteria at the genus level was shown using Circos (http://circos.ca/). Spearman correlations between specific bacterial genera, rumen fermentation parameters, and Dry matter digestibility was generated using the R program heatmap package. The sequences underlying the study is available in NCBI (PRJNA741061).

### Statistical Analysis

All groups were analyzed for DM digestibility and rumen fermentation parameters. The four groups st1su1 (Hl: 270 g/kg + 60 g/kg), st1su2 (Hh: 270 g/kg + 120 g/kg), st4su1 (Ll: 210 g/kg + 60 g/kg), and st4su4 (Lh: 210 g/kg + 120 g/kg) were further analyzed for the abundance of bacterial communities after fermentation for 24 h. All data were analyzed by two-way analysis of variance (ANOVA) using the General Linear Model Repeated Measure of SPSS software version 22.0. Data were analyzed using the model: *Y*_ijk_ = μ+ *F*_i_ + *V*_j_ + *F*_i_×*V*_j_ + *e*_ijk_, where μ is the overall mean, *F*_i_ is the effect of starch (*i* = 1–4), *V*_j_ is the effect of sugar (*j* = 1–4), *F*_i_ × *V*_j_ is starch × sugar level interaction, and *e*_ijk_ is the residual effect. Duncan contrasts were used to test the significance level for the effects of starch, sugar, and their interactions (starch × sugar), with *P* < 0.05 defined as statistical significance. The rumen fermentation parameters values were subjected to model curve fitting using origin 2018 software. At the same time, each parameter value in these model equations was calculated and the theoretical maximum yield, theoretical inflection point (maximum generation rate), and time for theoretical inflection point were calculated.

## Results

### Analysis of Rumen Fermentation Parameters

The effects of starch and sugar on rumen fermentation parameters are presented in Tables 1–3. DM digestibility was affected by sugar (*P* < 0.01) and the interaction of starch and sugar (*P* < 0.01). pH was significantly affected by starch (*P* < 0.01). Gas production was significantly affected by starch in the later period of culture and by sugar after 3–24 h of cultivation. Acetic acid was significantly affected by starch after 1–3 h and 12–24 h *in vitro* and by the interaction between starch and sugar. Propionic acid was significantly affected by starch at 1–6 and 24 h *in vitro*, and was significantly affected by sugar at 6 h *in vitro*. Both butyric acid and total volatile fatty acid (TVFA) contents were significantly affected by sugar after 1–24 h *in vitro*. TVFA was significantly affected by starch in the early stage of culture (0.5–6 h). The ratio of acetic to propionic acid (A/P ratio) was significantly affected by starch after 6 h and by sugar after 6–12 h.

**Table 2:** Comparison of VFA concentration (mmol/L) in each group.

**Item**	**Treatments**																**SEM**	***P*** **-value**		
	**St1 × su1**	**St2 × su1**	**St3 × su1**	**St4 × su1**	**St1 × su2**	**St2 × su2**	**St3 × su2**	**St4 × su2**	**St1 × su3**	**St2 × su3**	**St3 × su3**	**St4 × su3**	**St1 × su4**	**St2 × su4**	**St3 × su4**	**St4 × su4**		**Starch**	**Sugar**	**St × su**
**Acetic acid**																				
0.5	12.87	12.99	13.28	14.24	13.74	13.66	13.40	13.87	14.05	13.31	14.13	14.20	13.69	14.02	13.31	15.57	0.13	0.12	<0.05	0.42
1	13.89	15.20	15.51	15.01	15.08	13.85	15.41	14.96	15.30	14.90	15.08	15.21	15.04	16.36	15.42	16.64	0.15	<0.05	0.39	0.26
3	14.74	16.37	15.63	15.30	16.92	15.50	18.19	15.85	16.83	16.73	17.65	18.54	16.01	16.80	18.09	18.34	0.20	<0.01	<0.01	<0.01
6	32.66	37.89	35.40	36.05	35.68	30.31	34.35	36.27	35.87	40.62	35.34	34.63	33.78	36.23	40.25	39.79	0.56	0.12	0.42	0.06
12	39.44	39.28	38.20	38.76	38.29	35.65	39.36	41.62	40.51	42.71	45.22	40.14	41.67	42.55	43.10	42.34	0.49	<0.01	0.59	0.35
24	42.37	45.11	45.87	40.56	45.52	45.21	45.11	44.23	47.70	42.48	47.33	42.17	52.35	56.54	51.22	48.24	0.74	<0.01	0.08	0.48
**Propionic acid**																				
0.5	2.54	2.53	2.64	2.79	2.68	2.65	2.58	2.67	2.77	2.58	2.74	2.78	2.67	2.74	2.56	2.83	0.79	0.82	0.38	0.92
1	4.88	5.79	5.66	5.74	5.37	5.27	6.11	5.58	5.91	5.76	5.81	5.84	6.07	6.16	5.97	6.62	0.67	<0.01	0.26	0.37
3	5.84	5.83	6.19	6.04	5.67	6.49	6.30	6.46	6.47	6.38	7.31	6.15	6.39	6.71	7.19	7.26	0.74	<0.05	0.12	0.69
6	8.05	10.31	10.80	10.56	10.23	9.30	11.53	11.46	11.89	14.73	12.97	14.44	8.50	12.50	13.93	12.89	1.80	<0.01	<0.01	0.39
12	14.98	15.21	14.44	14.01	14.00	13.24	16.28	14.93	15.16	16.60	16.22	15.22	15.14	15.18	14.26	14.07	1.74	0.07	0.50	0.16
24	20.25	21.69	21.67	18.77	22.11	22.42	21.15	21.96	28.95	20.97	22.00	20.79	26.63	28.24	23.92	22.80	1.67	<0.01	0.06	0.16
**Butyric acid**																				
0.5	0.95	0.92	0.97	1.03	1.00	0.99	0.96	1.02	1.04	0.92	0.95	1.01	0.92	0.94	0.87	1.03	0.01	0.18	0.66	0.917
1	1.18	1.59	1.32	1.22	1.17	1.12	1.16	1.13	1.19	1.15	1.14	1.12	1.21	1.24	1.29	1.32	0.02	0.19	<0.01	0.818
3	2.15	2.87	2.62	1.75	1.53	2.17	1.73	1.41	2.49	2.34	2.42	2.01	2.24	3.52	4.52	4.97	0.16	0.70	<0.01	0.948
6	4.01	3.55	4.37	4.25	5.15	5.12	3.97	4.96	4.44	5.12	4.89	3.34	4.55	4.81	7.43	6.13	0.15	<0.01	<0.01	0.945
12	6.27	4.62	5.81	5.97	6.71	6.13	6.01	5.18	5.58	5.62	6.93	5.50	5.94	5.42	8.07	8.49	0.17	0.84	<0.01	0.42
24	7.06	6.34	6.63	6.71	7.40	7.49	7.27	7.35	9.89	7.49	9.03	7.33	9.87	11.50	10.33	9.00	0.26	<0.01	<0.01	0.96

**Table 3:** Comparison of TVFA concentration (mmol/L) and ratio of acetic acid to propionic acid (A/P) in each group.

**Item**	**Treatments**																**SEM**	***P*** **-value**		
	**St1 × su1**	**St2 × su1**	**St3 × su1**	**St4 × su1**	**St1 × su2**	**St2 × su2**	**St3 × su2**	**St4 × su2**	**St1 × su3**	**St2 × su3**	**St3 × su3**	**St4 × su3**	**St1 × su4**	**St2 × su4**	**St3 × su4**	**St4 × su4**		**Starch**	**Sugar**	**St × su**
**TVFA**																				
0.5	16.36	16.45	16.90	18.07	17.42	17.30	16.94	17.56	17.85	16.81	17.81	17.98	17.29	17.71	16.74	19.43	0.31	<0.05	0.27	0.57
1	19.95	22.59	22.49	21.97	21.61	20.24	22.68	21.67	22.40	21.80	22.04	22.18	22.33	23.76	22.68	24.58	0.53	<0.29	<0.05	0.22
3	22.73	25.08	24.44	23.09	24.12	24.16	26.21	23.72	25.79	25.46	27.38	26.69	24.64	27.02	29.80	30.57	0.64	<0.01	<0.01	<0.05
6	44.73	51.75	50.57	50.86	51.06	44.73	49.85	52.68	52.19	60.48	53.19	52.41	46.83	53.55	61.61	58.82	1.27	<0.05	<0.01	<0.05
12	60.69	59.11	58.45	58.74	58.99	55.01	61.65	61.74	61.24	64.93	68.37	60.86	62.76	63.15	65.43	64.91	1.59	0.33	<0.01	0.41
24	69.68	73.15	74.17	66.05	75.03	75.12	73.54	73.53	86.54	70.95	78.36	70.29	88.85	96.28	85.47	80.04	2.29	0.06	<0.01	0.19
**A/P ratio**																				
0.5	5.10	5.14	5.03	5.10	5.12	5.16	5.20	5.20	5.08	5.16	5.16	5.12	5.13	5.13	5.19	5.61	0.04	0.52	0.40	0.76
1	2.85	2.62	2.74	2.62	2.90	2.64	2.52	2.69	2.59	2.59	2.60	2.61	2.48	2.66	2.59	2.52	0.03	0.51	0.15	0.38
3	2.52	2.81	2.52	2.55	2.98	2.39	2.89	2.47	2.73	2.65	2.41	3.05	2.53	2.50	2.52	2.53	0.04	0.70	0.29	<0.05
6	4.15	3.69	3.28	3.42	3.52	3.23	3.13	3.18	3.07	2.77	2.73	2.43	3.43	2.90	2.93	3.16	0.10	<0.05	<0.05	0.80
12	2.63	2.58	2.65	2.77	2.73	2.73	2.47	2.79	2.67	2.58	2.79	2.64	2.75	2.81	3.02	3.03	0.03	0.49	<0.05	0.58
24	2.09	2.08	2.12	2.16	2.06	2.02	2.13	2.01	1.76	2.08	2.15	2.03	1.97	2.01	2.14	2.11	0.02	0.06	0.28	0.54

### Model Fitting

**Figure 1:**
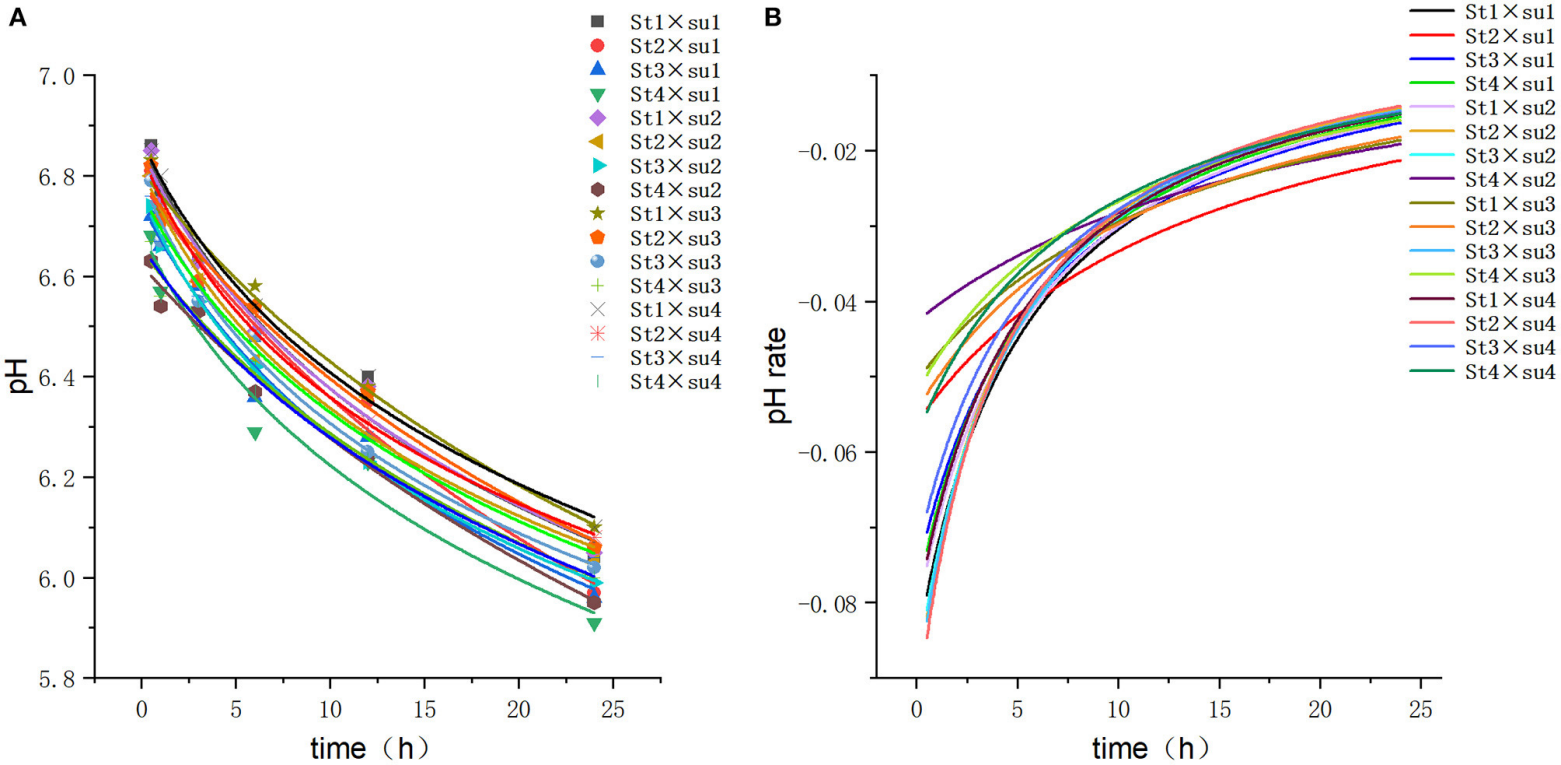
Fitting model curve of pH. **(A)** Logarithmic model fitting curve. **(B)** Derivative of Logarithmic model.

**Figure 2:**
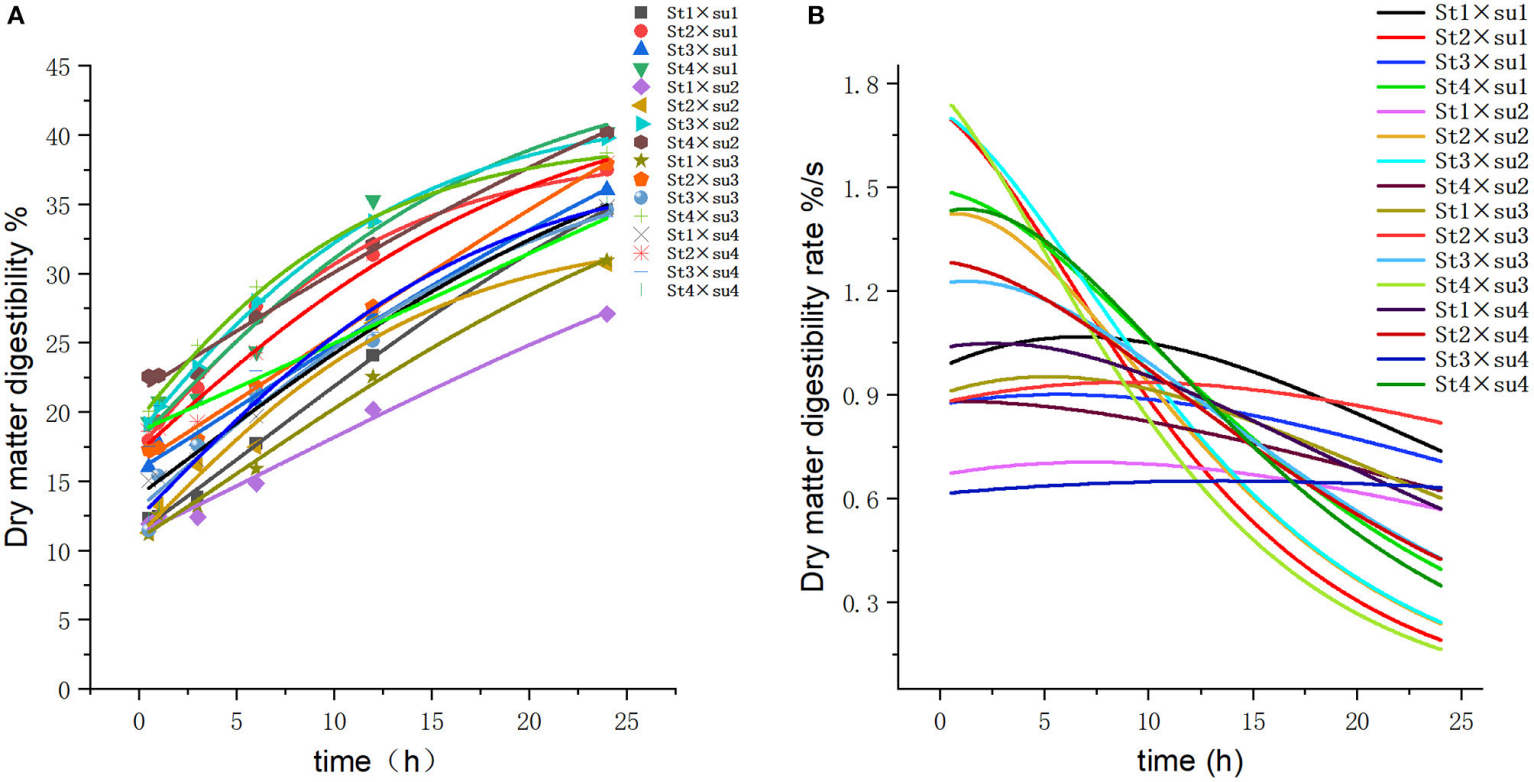
Fitting model curve of DM digestibility. **(A)** Gompertz model fitting curve. **(B)** Derivative of Gompertz.

**Figure 3:**
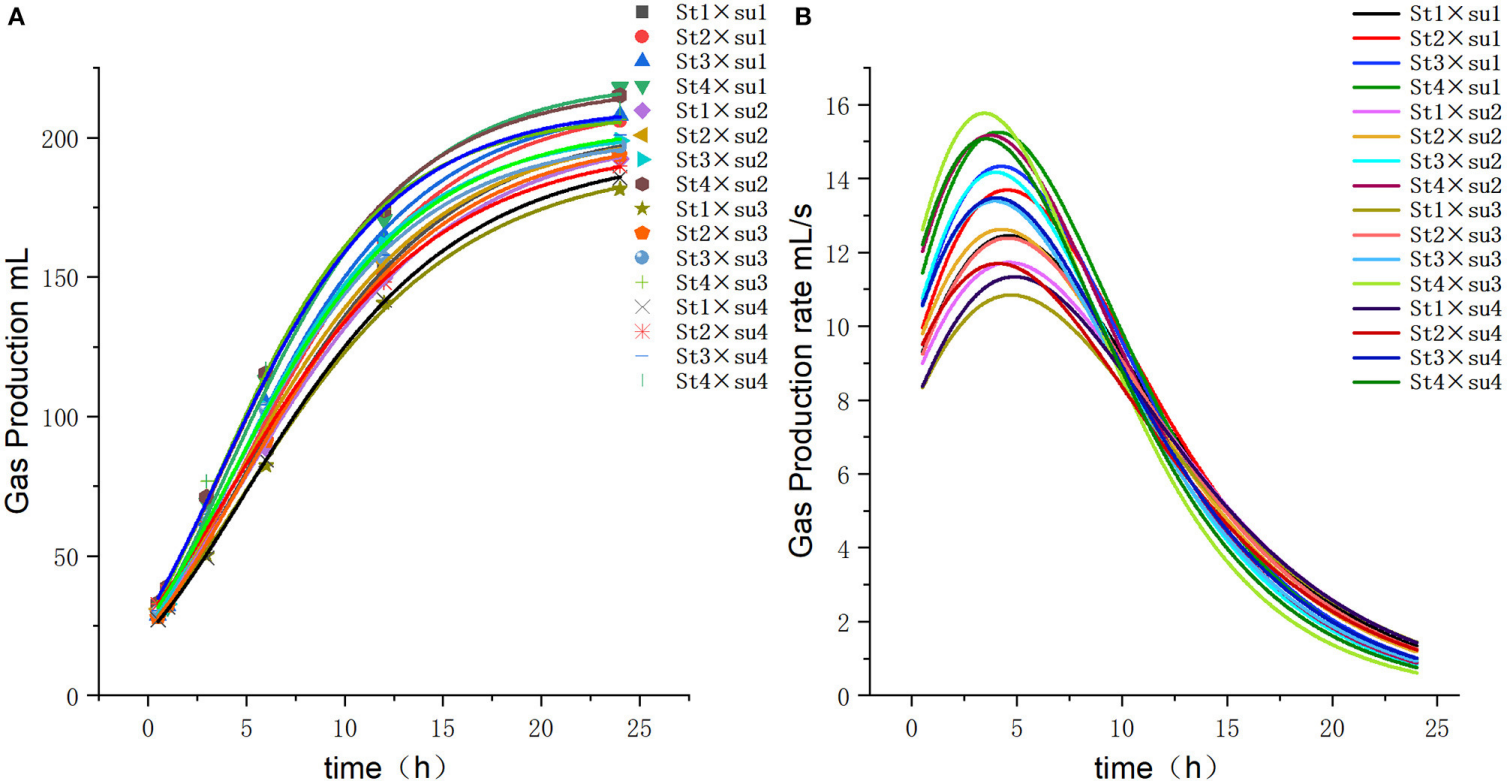
Fitting model curve of gas production. **(A)** Gompertz model fitting curve. **(B)** Derivative of Gompertz.

**Figure 4:**
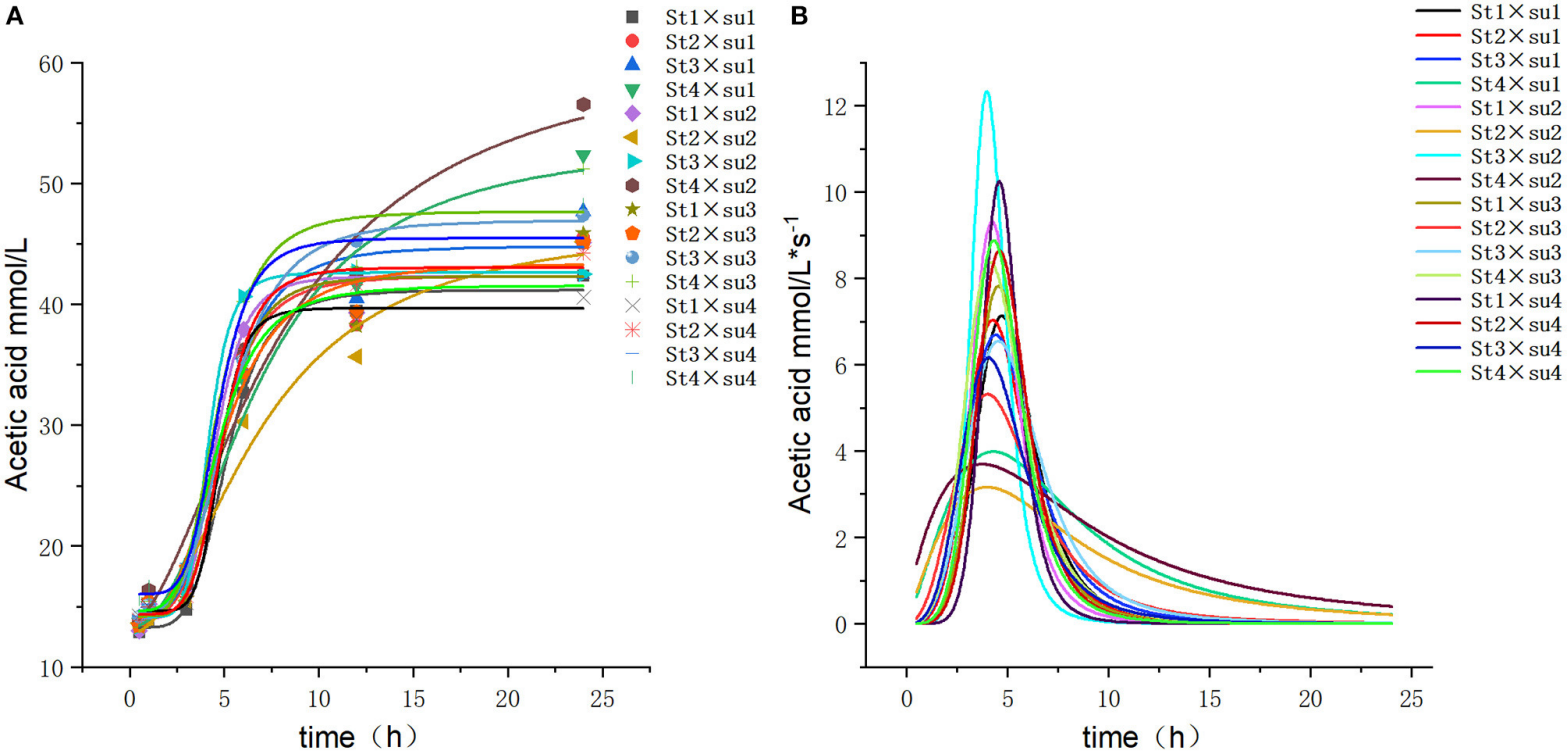
Fitting model curve of acetic acid production. **(A)** Logistic model fitting curve. **(B)** Derivative of Logistic.

**Figure 5:**
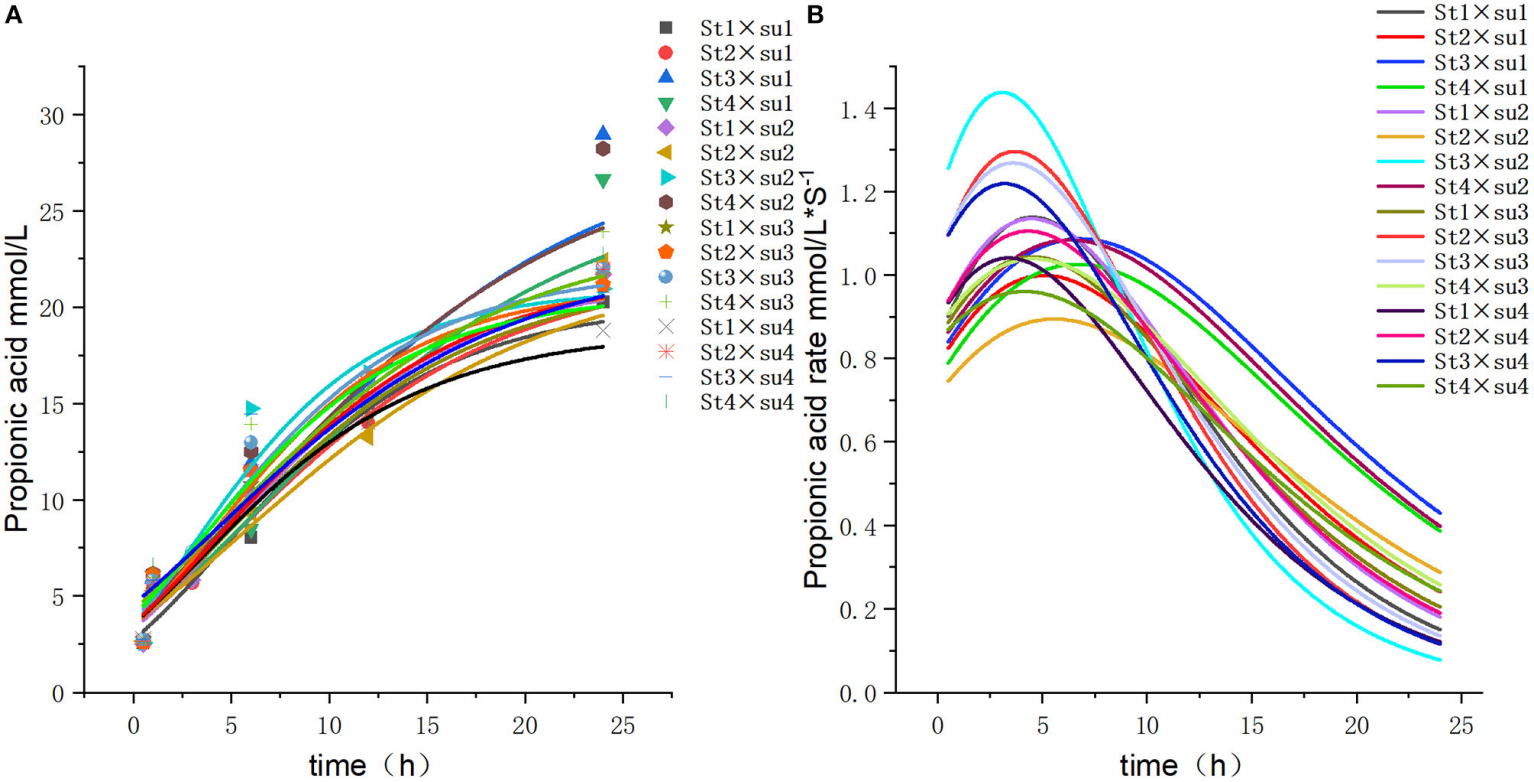
Fitting model curve of propionic acid production. **(A)** Gompertz model fitting curve. **(B)** Derivative of Gompertz.

**Figure 6:**
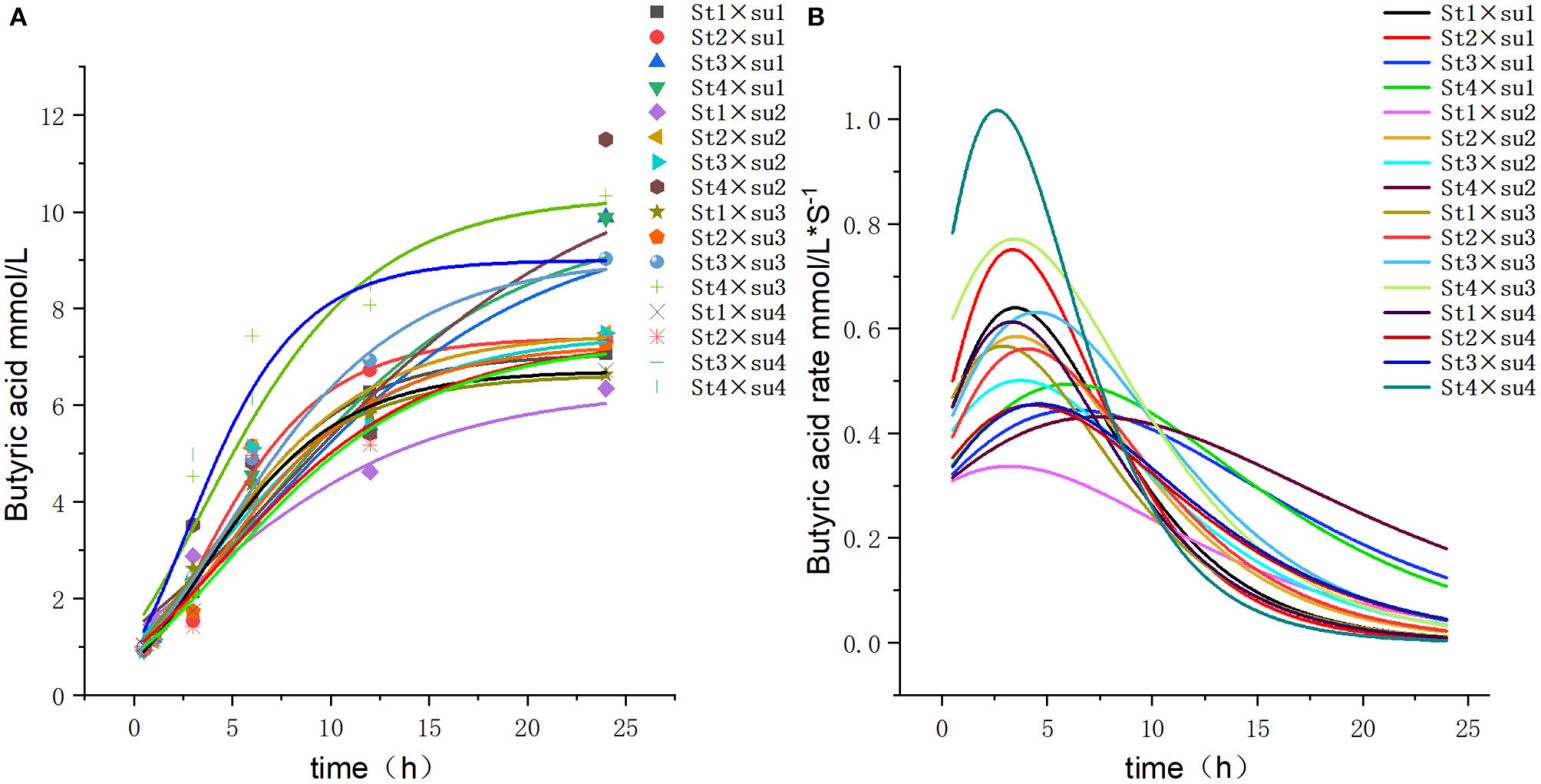
Fitting model curve of butyric acid production. **(A)** Gompertz model fitting curve. **(B)** Derivative of Gompertz.

**Figure 7:**
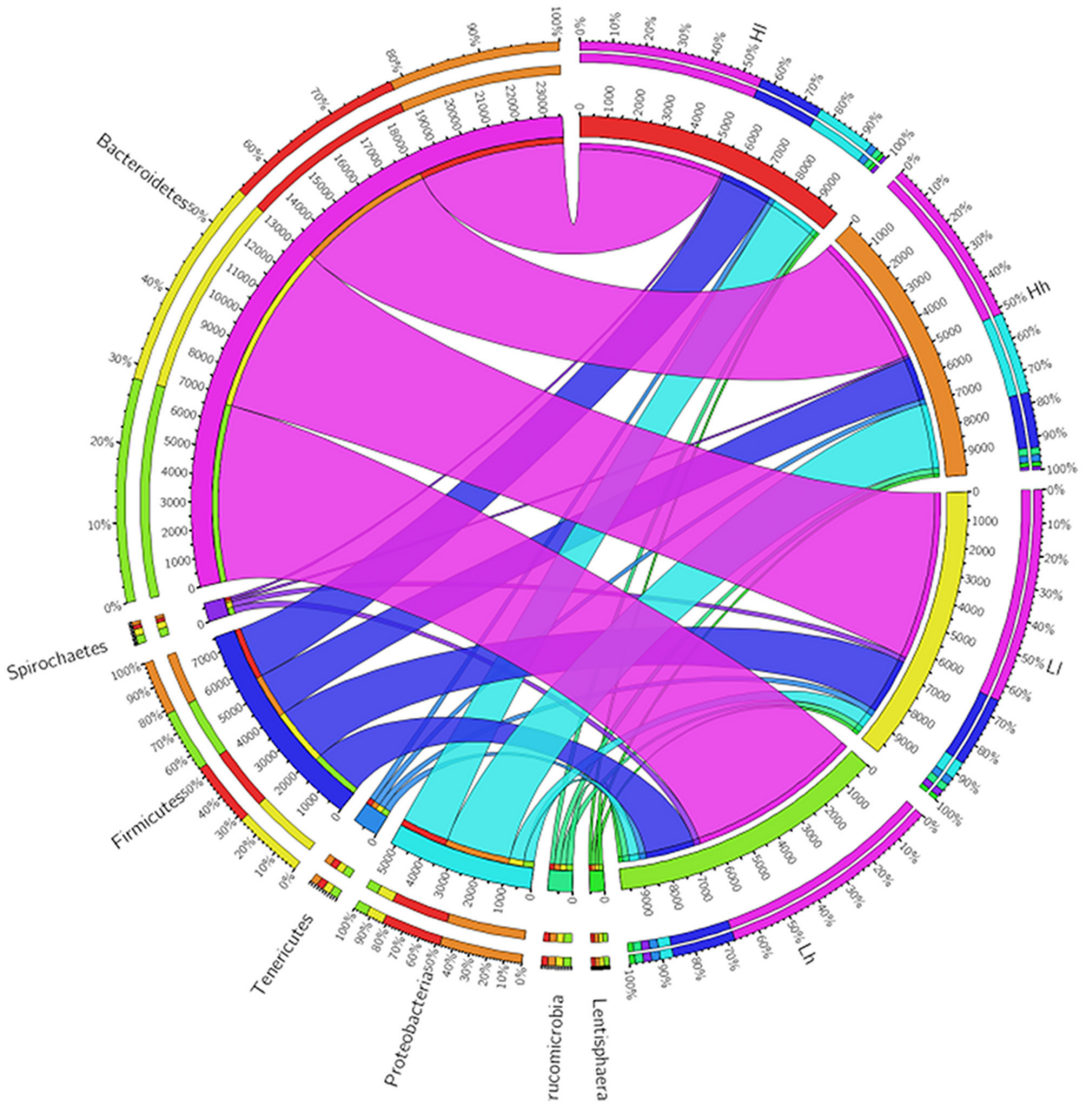
Circos showed Bacterial phyla of 4 the groups.

**Figure 8:**
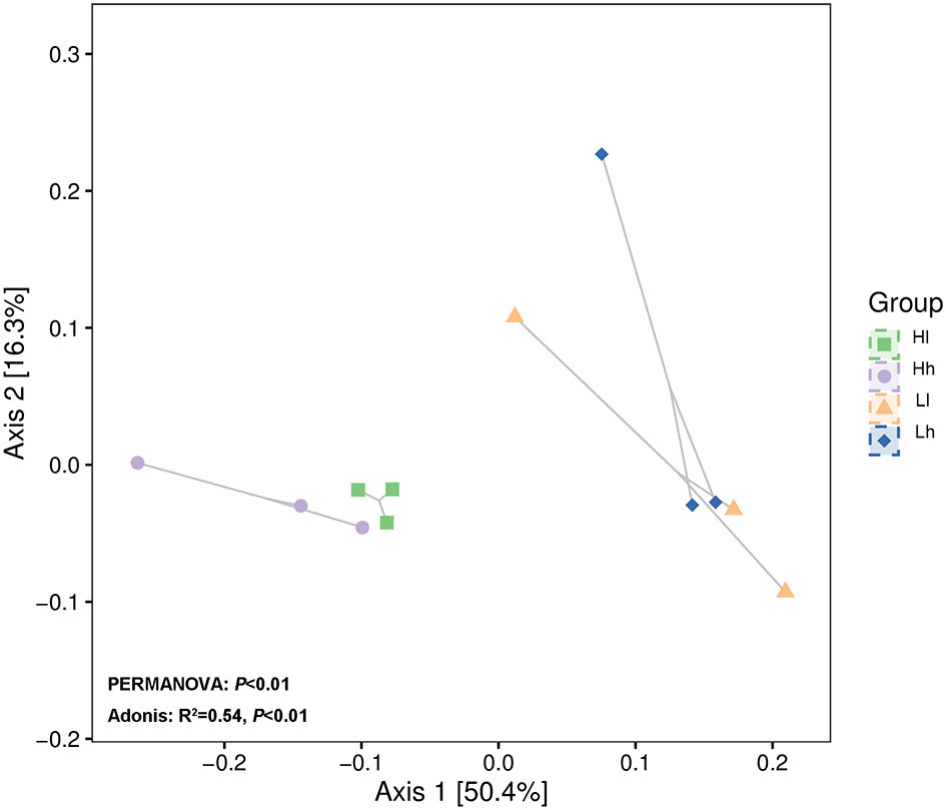
Principal coordinates analysis (PCoA) revealing bacterial genera in groups with different combinations of sugar and starch levels based on Bray–Curtis (Hl, Diet of high starch and low sugar; Lh, Diet of low starch and high sugar; Hh, Diet of high starch and high sugar).

These results indicated that these three equations have high fitting degrees (*R*^2^ > 0.83; Supplementary Tables 3–5); the model curves are shown in Figures 1–**8**. Specifically, the accumulation amounts of fermentation index differed between groups at 24 h. Moreover, the maximum production rate and its production time also differed among groups with similar accumulation amounts. When the starch concentration in the diet was 25%, the accumulation of acetic acid among the groups with different sugar content was similar, but the maximum production rate differed by 3–4-fold. Increasing the sugar concentration in the diet could increase the production rate of fermentation products and advance the time of maximum production rate.

### Rumen Bacterial Community Composition

Table 4 presents the Chao1, Simpson, and Shannon indices; these were not significantly affected by starch, sugar, or the starch × sugar interaction. Notably, the Simpson and Shannon indices were higher in the Ll diet than in the Hh diet groups (*P* < 0.05).

**Table 4:** Effects of different combinations of sugar and starch on the alpha diversity of ruminal bacterial communities *in vitro*.

**Item**	**Starch level**		**Sugar level**		**Group** ^**1**^ **%**				**SEM**	***P*** **-value**		
	**H**	**L**	**h**	**l**	**Ll**	**Lh**	**Hl**	**Hh**		**Starch**	**Sugar**	St*Su
OTU	2,865	3,154	3,057	2,962	1,487	1,677	1,475	1,390	68.23	0.34	0.75	0.38
Chao1	1,285.92	1,376.88	1,358.94	1,303.86	1,304.58	1,449.17	1,303.14	1,268.71	151.87	0.34	0.56	0.35
Simpson	0.970	0.974	0.969	0.975	0.976^b^	0.972^ab^	0.971^ab^	0.967^a^	0.005	0.10	0.05	0.83
Shannon	7.01	7.29	7.04	7.26	7.36^b^	7.20^ab^	7.15^ab^	6.87^a^	0.26	0.06	0.13	0.64

a,b *Means in the same row with different superscripts are significantly different (P < 0.05). H, Diet of high starch; L, Diet of low starch; h, Diet of high sugar; l, Diet of low sugar; Ll, Diet of low starch and low sugar; Lh, Diet of low starch and high sugar; Hl, Diet of high starch and low sugar; Hh, Diet of high starch and low sugar*.

#### Bacterial Relative Abundance

Amplicon sequencing of the partial 16S rDNA gene generated a total of 1,234,738 high-quality sequences and an average of 102,894 sequences per sample, which were assigned to 6,019 OTUs. Taxonomic analysis identified that the sequences belonged to 22 bacterial phyla and 97 bacterial genera. The predominant bacterial phyla encompassed 10 taxa (with the relative abundance being > 0.10% in each sample), with Bacteroidetes (50.02%), Firmicutes (18.86), Proteobacteria (12.92%), Tenericutes (2.30%), and Verrucomicrobia (2.18%) being the most abundant. The predominant bacterial taxa at the genus level (with relative abundance being > 0.10% in each sample) consisted of 34 genera, with *unidentified_ Bacteroidales* (20.57%), *Prevotella* (20.17%), *Ruminobacter* (10.54%), *CF231* (8.15%), and *Ruminococcus* (3.12%) being the most abundant (Table 5).

**Table 5:** Comparison of rumen bacterial genera in each group (%).

**Phylum**	**Genus**	**Starch level**		**Sugar level**		**Group** ^**1**^ **%**				**SEM**	***P*** **-value**		
		**H**	**L**	**h**	**l**	**Ll**	**Lh**	**Hl**	**Hh**		**Starch**	**Sugar**	St*Su
*Bacteroidetes*		59.30	58.75	65.15	52.90	50.99^a^	66.51^b^	54.81^a^	63.78^b^	2.15	0.82	<0.01	0.19
	*Prevotella*	20.04	20.31	20.39	19.96	19.53	20.09	20.39	19.68	3.18	0.90	0.84	0.60
	*unidentified*_*Bacteroidales*	19.75	21.38	23.35	17.78	17.4^a^	25.2^b^	18.0^a^	21.4^b^	4.15	0.38	<0.05	0.24
	*CF231*	8.15	8.14	8.18	8.12	8.08	8.21	8.27	8.03	1.51	0.99	0.96	0.86
	*BF311*	5.37	4.21	7.50	2.08	1.23^a^	7.19^b^	2.94^a^	7.81^b^	3.27	0.29	<0.01	0.61
	*BS11*	2.19	1.67	2.13	1.73	1.62	1.73	1.84	2.54	0.69	0.23	0.34	0.48
	*unclassified_Bacteroidales*	1.00	0.71	0.98	0.73	0.62	0.81	0.84	1.15	0.32	0.14	0.19	0.75
	*unidentified_Prevotellaceae*	0.78	0.55	0.71	0.63	0.54	0.57	0.71	0.84	0.25	0.16	0.58	0.75
	*unidentified_[Paraprevotellaceae]*	1.14	0.84	1.19	0.79	0.71^a^	0.97^ab^	0.87^ab^	1.41^b^	0.39	0.16	0.07	0.49
	*unidentified_S24-7*	0.26	0.14	0.26	0.14	0.11^a^	0.17^ab^	0.17^ab^	0.35^b^	0.14	0.11	0.10	0.38
	*unidentified_RF16*	0.25	0.18	0.22	0.21	0.17	0.18	0.25	0.25	0.08	0.15	0.94	0.87
*Firmicutes*		20.28	17.44	19.82	17.91	16.32^a^	18.57^ab^	19.50^ab^	21.07^b^	0.67	<0.05	0.10	0.75
	*Ruminococcus*	3.94	2.30	3.23	3.01	2.34^a^	2.27^a^	3.68^b^	4.20^b^	0.93	<0.01	0.29	0.17
	*unidentified_Clostridiales*	2.66	2.69	2.80	2.55	2.37	3.01	2.73	2.59	0.41	0.90	0.29	0.12
	*unidentified_Lachnospiraceae*	2.23	2.15	2.34	2.04	1.91	2.39	2.17	2.29	0.42	0.78	0.27	0.50
	*unidentified_Ruminococcaceae*	2.09	2.14	2.15	2.08	2.00	2.29	2.16	2.01	0.36	0.81	0.77	0.35
	*Butyrivibrio*	2.07	2.16	2.29	1.93	0.21	2.23	1.79	2.34	0.72	0.85	0.47	0.68
	*Streptococcus*	0.51	0.44	0.29	0.66	0.62	0.26	0.70	0.33	0.30	0.65	<0.05	0.99
	*Clostridium*	0.88	0.78	0.79	0.86	0.75	0.80	0.98	0.78	0.22	046	0.62	0.37
	*Anaerovibrio*	0.90	0.71	0.88	0.72	0.70	0.72	0.74	1.05	0.34	0.39	0.45	0.51
	*unidentified_Veillonellaceae*	0.71	0.57	0.66	0.61	0.60	0.55	0.63	0.78	0.15	0.12	0.53	0.23
	*unclassified_Lachnospiraceae*	0.52	0.44	0.47	0.49	0.43	0.45	0.55	0.49	0.08	0.10	0.66	0.38
	*Selenomonas*	0.54	0.26	0.52	0.28	0.15	0.37	0.40	0.68	0.35	0.21	0.25	0.90
	*unidentified_[Mogibacteriaceae]*	0.38	0.28	0.37	0.29	0.23	0.32	0.34	0.42	0.14	0.22	0.34	0.91
	*Succiniclasticum*	0.31	0.23	0.25	0.28	0.24^ab^	0.22^a^	0.33^b^	0.28^ab^	0.07	<0.05	0.30	0.69
	*Oscillospira*	0.24	0.25	0.24	0.25	0.25	0.25	0.26	0.23	0.02	0.80	0.64	0.37
	*unclassified_Clostridiales*	0.25	0.23	0.28	0.20	0.18^a^	0.29^b^	0.22^a^	0.28^b^	0.06	0.35	<0.05	0.14
	*Pseudobutyrivibrio*	0.32	0.19	0.30	0.21	0.17^a^	0.21^ab^	0.26^ab^	0.38^b^	0.11	<0.05	0.15	0.43
	*p-75-a5*	0.14	0.16	0.15	0.15	0.14^ab^	0.19^b^	0.16^ab^	0.12^a^	0.04	0.27	0.95	<0.05
*Proteobacteria*		11.63	14.21	4.62	21.23	24.50^b^	3.93^a^	17.96^b^	5.30^a^	2.85	0.37	<0.01	0.18
	*Ruminobacter*	9.54	11.56	2.99	18.11	20.9^b^	2.16^a^	15.2^b^	3.81^a^	9.33	0.52	<0.05	0.26
	*Acinetobacter*	0.82	1.26	0.55	1.54	1.78^b^	0.75^ab^	1.30^ab^	0.35^a^	0.78	0.26	<0.05	0.91
	*Desulfovibrio*	0.37	0.25	0.30	0.32	0.28	0.21	0.36	0.38	0.11	0.07	0.66	0.44
	*Succinivibrio*	0.30	0.30	0.24	0.36	0.39	0.22	0.33	0.27	0.11	0.93	0.09	0.38
*Tenericutes*		2.25	2.35	2.48	2.12	2.10	2.60	2.14	2.37	0.15	0.79	0.31	0.70
	*unidentified_RF39*	1.90	2.00	2.14	1.76	1.78	2.23	1.74	2.06	0.49	0.75	0.24	0.83
	*unidentified_ML615J-28*	0.31	0.30	0.29	0.31	0.28	0.32	0.35	0.27	0.07	0.78	0.62	0.18
*Verrucomicrobia*		2.02	2.33	2.37	1.98	2.21	2.46	1.76	2.29	0.21	0.52	0.42	0.78
	*unidentified_RFP12*	1.84	2.11	2.14	1.80	2.01	2.21	1.60	2.07	0.73	0.58	0.49	0.78
*Lentisphaerae*		1.19	1.45	1.46	1.19	1.29	1.61	1.08	1.31	0.11	0.27	0.25	0.85
	*unidentified_Victivallaceae*	1.18	1.43	1.44	1.17	1.28	1.58	1.06	1.29	0.37	0.28	0.25	0.87
*Spirochaetes*		1.56	1.75	2.28	1.03	1.03^a^	2.46^b^	1.04^a^	2.09^ab^	0.11	0.64	<0.05	0.63
	*Sphaerochaeta*	0.31	0.46	0.36	0.40	0.45^ab^	0.47^b^	0.36^ab^	0.26^a^	0.04	<0.05	0.518	0.364
	*Treponema*	1.01	0.97	1.49	0.49	0.47	1.46	0.50	1.52	0.22	0.91	<0.05	0.97

1 *Group %: H, Diet of high starch; L, Diet of low starch; h, Diet of high sugar; l, Diet of low sugar; Ll, Diet of low starch and low sugar; Lh, Diet of low starch and high sugar; Hl, Diet of high starch and low sugar; Hh, Diet of high starch and low sugar*.

a,b *Means in the same row with different superscripts are significantly different. (P < 0.05)*.

At the phylum level, the relative abundance of Bacteroidetes, Proteobacteria, and Spirochaetes was significantly influenced by sugar, and the levels of Bacteroidetes and Spirochaetes in Hh and Lh were higher than those in Ll and Hl groups, whereas the reverse was true for the relative abundance of Proteobacteria (Figure 7). At the genus level, the relative abundance of *Ruminococcus* and *Pseudobutyrivibrio* was affected by starch (*P* < 0.05), with *Ruminococcus* being the prominent genus in the H starch groups. Sugar had effects on the relative abundance of *BF311, unidentified_Bacteroidales, Streptococcus, unclassified_Clostridiales, Ruminobacter*, and *Acinetobacter* (*P* < 0.05). Furthermore, *unidentified_Bacteroidales1, Oscillospira*, and *BF31* were distinguished as prominent genera in H sugar groups, while the relative abundance of *Ruminobacter* was the lowest in these groups. The relative abundances of both *p-75-a5* and *Sphaerochaeta* were affected by the interaction of starch and sugar. Furthermore, the relative abundance of *unidentified_[Paraprevotellaceae], unidentified_S24-7*, and *Pseudobutyrivibrio* was higher in the Hh group than in the Ll group (*P* < 0.05), while that of *Acinetobacter* was significantly lower in Hh group than in the Ll group (*P* < 0.05).

#### Beta Diversity

Patterns of variation in the bacterial community composition were observed among groups with different starch and sugar levels using principal coordinates analysis (PCoA). Analysis of similarity based on Bray-Curtis dissimilarity showed significant difference in bacterial clusters between the 4 groups (Figure 8, PERMANOVA: *P* < 0.01; Adonis: *R*^2^ = 0.54, *P* < 0.01). Analysis of similarity also showed that bacterial clusters in the L and H starch groups were distinct, but there were some overlaps in the l and h sugar groups. The results indicated that starch content may have a greater impact on bacterial clusters than the sugar content in the diets.

### The Relationship Between Rumen Fermentation Parameters and Microbiota

**Figure 9:**
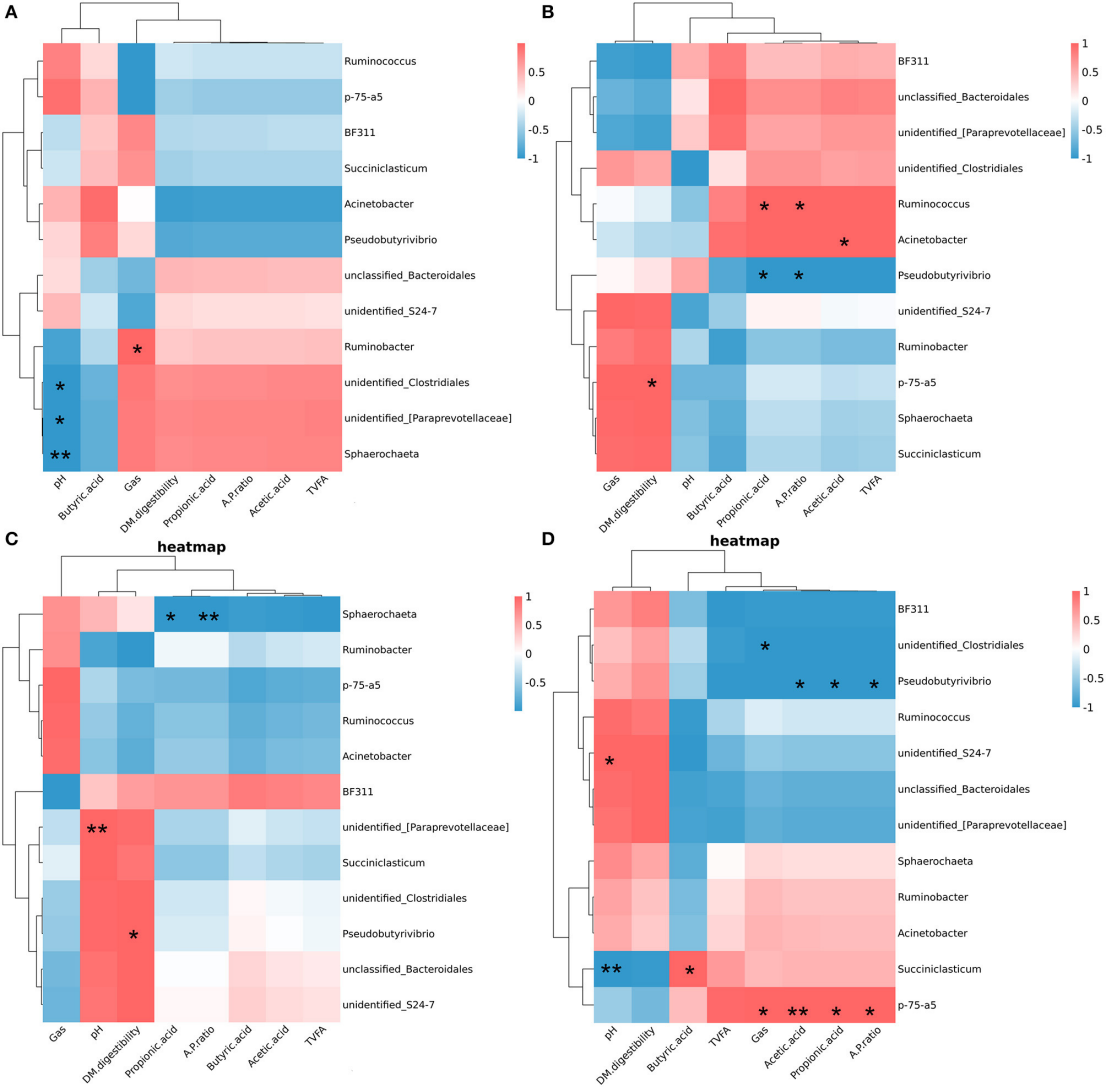
Spearman correlations between specific bacterial genera, rumen fermentation parameters, and Dry matter digestibility. **(A)** Spearman correlations within diet of diet of low starch and low sugar. **(B)** Spearman correlations within diet of diet of low starch and high sugar. **(C)** Spearman correlations within diet of diet of high starch and low sugar. **(D)** Spearman correlations within diet of diet of high starch and high sugar. ^*^P < 0.05; ^**^p < 0.01.

Pearson correlation analysis showed that the relationship between the relative abundances of the differential bacterial at the genus and rumen fermentation parameters within each group (Figure 9). In the Ll diet, pH was negatively correlated with the relative abundances of *unidentified_[Paraprevotellaceae]* (*P* < 0.05), *unclassified_Clostridiales*, and *Sphaerochaeta*, and gas production was positively correlated with the relative abundance of *Ruminobacter* (*P* < 0.05). In the Lh diet, DM digestibility was positively correlated with the relative abundance of *p-75-a5* (*P* < 0.05); acetic acid was positively correlated with the relative abundance of *Acinetobacter* (*P* < 0.05); propionic acid and A/P were positively correlated with the relative abundance of *Ruminococcus* (*P* < 0.05), which were negatively correlated with the relative abundances of *Pseudobutyrivibro* (*P* < 0.05). In the Hl diet, pH was positively correlated with the relative abundances of *unidentified_[Paraprevotellaceae]* (*P* < 0.05), DM digestibility was positively correlated with the relative abundance of *Pseudobutyrivibro* (*P* < 0.05). And propionic acid and A/P were negatively correlated with the relative abundance of *Sphaerochaeta* (*P* < 0.05). In the Hh diet, the relative abundance of *p-75-a5* was positively correlated with gas production, acetic acid, propionic acid and A/P (*P* < 0.05); the relative abundances of *Pseudobutyrivibro* were positively correlated with acetic acid, propionic acid and A/P (*P* < 0.05). The relative abundances of *Succinictasticum* was positively correlated with butyric acid (*P* < 0.05), but were negatively correlated with pH (*P* < 0.05).

### Differential Functions of the Rumen Microbiome Among Different Combinations of Sugar and Starch

**Figure 10:**
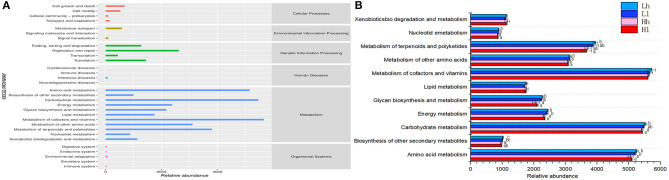
**(A)** Predicted KEGG secondary functional pathway abundance map. The abscissa is the abundance of the functional pathway (unit: KO per million), the ordinate is the functional pathway of the second classification level of KEGG, and the rightmost is the first-level pathway to which the pathway belongs. Shown here is the average abundance of all samples. **(B)** Comparison of rumen microbial KEGG. Significantly different modules in each significantly different level 2 pathway (Lh, light blue; Ll, navy blue; Hh, pink; Hl, red) were presented.

The KEGG pathway database is a biological metabolic pathway analysis database. Based on KEGG analysis profiles, 11 endogenous second-level pathways belonged to the following the first-level categories “Metabolism” were considered as potential rumen microbial metabolic pathways (Figure 10).

In the six first-level categories, rumen microbial metabolic pathways were mainly enriched in the “Metabolism” category, with Metabolism of cofactors and vitamins (5,657.03 KO/million), Carbohydrate metabolism (5,463.19 KO/million), Amino acid metabolism (5,152.41 KO/million), Metabolism of terpenoids and polyketides (3,802.71 KO/million), and Metabolism of other amino acids (3,110.50 KO/million) being the most abundant. The second level categories all belonged to “Metabolism.” These pathways in the Metabolism among the four groups were further analyzed and found that the relative abundance of Amino acid metabolism, Energy metabolism, Glycan biosynthesis and metabolism, Metabolism of other amino acids, Nucleotide metabolism in the Ll and Lh were significantly high than those in the Hl and Hh (*P* < 0.05). The relative abundance of Biosynthesis of other secondary metabolites in Lh was higher than that of Hh (*P* < 0.05). And the relative abundance of Metabolism of terpenoids and polyketides in Lh was higher than that of Hl (*P* < 0.05). The results revealed that different combinations of starch and sugar affected microbial metabolism, and sugar could increase metabolism, which showed that sugar could not completely replace the role of starch in rumen fermentation.

## Discussion

### Ruminal Fermentation

Altering the combination of starch and sugar is an essential adjustment to the energy structure of the diet, specifically the adjustment of non-fibrous carbohydrates. Because the metabolizable energy of starch and sugar is similar, the inter-replacement of sugar and starch leads to very little, if any, loss of energy associated with diet and ensures that other energy structures remain unchanged. However, the different chemical structures of starch and sugar may lead to some differences in their subsequent digestion and fermentation in the rumen; this might result in differences in production and growth in ruminants. In the current some studies, increasing the concentration of sugar in the diet increased the concentration of butyric acid in the rumen; the mechanism for this has also been studied and elucidated. Mansfield et al. (22) found that increasing the water soluble carbohydrates concentration in the diet can increase butyric acid concentration. Martel et al. (8) reported that the concentration of TVFAs and butyric acid can be increased by using sugar instead of starch in the diet. In an *in vivo* experiment, Malhi et al. (10) observed that injection of butyric acid into the rumen of goats increased the growth of rumen epithelium cells and the absorption capacity of VFAs.

Penner et al. (23) noted that an increase in rumen pH was positively related to the capacity of ruminal epithelial tissues to take up acetate and butyrate. Furthermore, Oba (5) speculated that the reason why the substitution of sugar for starch did not lead to a further decrease in rumen pH in the fermentation process may be the high butyric acid production. As a specific number of moles of hexose ferments to the same number of moles of butyrate, but double the number of moles of propionate or acetate (24), less H+ is produced from butyric acid than from acetic acid and propionic acid. In the present study, the pH value decreased with an increase in sugar level; this may be attributed to the *in vitro* nature of the experiment. Although the buffer solution has a definite buffering function on the short volatile fatty acid (SVFA) produced, there is a lack of factors for absorption of VFAs in the rumen, resulting in the low pH value and accumulation of VFAs in this experiment. Meanwhile, as a result of the higher absorption rate of butyrate than that of acetate and propionate (25), the production of butyrate *in vivo* is likely to be lower than that *in vitro* conditions (26). Therefore, actual fermentation characteristics might be better observed in *in vitro* studies. Even so, the actual effects of the interaction between sugar and starch in diets on physiological function of animal organism need further validation *in vivo*.

Based on the conclusions of previous *in vivo* experiments, diets associated with a low rate of VFA production, long time for accumulation, and a higher level of accumulation are more conducive to reversing the growth and health of animals. This is because the low rate of production will not cause too much pressure on the rumen to exceed the ability of the rumen to absorb and cause rumen acidosis, and the extension of the duration increases the production of VFAs and improves the efficiency of feed utilization.

### Ruminal Bacterial-Community Composition

The present study found that increasing the sugar content in the diet led to a significant increase in the DM disappearance rate. However, this may be caused by the solubility of sugar (5). Increased amount of nutrients might be associated with a higher likelihood of fermentation and digestion by the ruminal micro-ecosystem. Although the sugar had been fully utilized in the early stage of fermentation, the DM disappearance rate of the high-sugar group was still significantly higher than that of the low-sugar group in the late stage of fermentation.

The effect of sugars, such as sucrose, on ruminal microorganisms has not been extensively studied (26) despite the large number of microorganisms that colonize the rumen (34), participate in nutrient digestion, and play a vital role in ruminant fermentation. A total of 17 phyla and 300 bacterial genera were detected in this study, of which *Bacteroidetes, Firmicutes*, and *Proteobacteria* were the most dominant phyla in the rumen bacterial communities. These results are consistent with those of previous studies (27), which also showed that changing sugar and starch content in the diet does not change the dominant bacterial communities in the rumen. In the present study, the abundance of *Bacteroidetes* in the Lh group was significantly higher than that in the other three groups. *Bacteroidetes* is a gram-negative bacterium and a major bacterium that digests and decomposes NSC. Some of its genes are closely related to the genes of carbohydrate metabolism. Some studies have shown that feeding high-concentration diets to dairy cows reduced the relative abundance of *Bacteroidetes* in the rumen (28, 29), because the resulting low pH value may inhibit the growth of gram-negative bacteria (30). We found that the high-concentration diets by increasing sugar level did not reduce the relative abundance of the *Bacteroides* phylum. On the contrary, it significantly increased the relative abundance of *Bacteroides*. This may be because the high sugar did not lead to a sharp drop in ruminal pH, with a consequent protective effect on *Bacteroides*.

*Proteobacteria* is one of the main bacterial phyla in the rumen of ruminants. A positive correlation was noted between the abundance of *Proteobacteria* and sugar/starch concentration in the diet. Both *Ruminobacter* and *Desulfovibrio* belong to the *Proteobacteria* phylum. *Desulfovibrio* is a strictly anaerobic gram-negative bacterium that competes with *Firmicutes* for carbon sources and energy substances, and its metabolites can promote the secretion of interleukin-6 and interleukin-8, and thereby induce an inflammatory response (31, 32). The current study showed that increasing sugar and starch levels did not raise the relative abundance of *Desulfovibrio*, which may not be harmful to health of rumen and intestine of ruminants. There has been less research on *Desulfovibrio* in the rumen; thus, it remains to be determined whether it is strongly related to the health of ruminants. *Ruminobacter amylophilus*, another member of *Proteobacteria*, is associated starch fermentation; it also plays a role in protein digestion. Our data revealed that high-sugar diets significantly reduced the relative abundance of *Proteobacteria*.

Starch is mainly digested by *Streptococcus*, whose fermentation product is lactic acid. Results on the effect of dietary sugar levels on the relative abundance of *Streptococcus* are equivocal: De Souza et al. (33) observed that increased sugar levels raised the abundance of *Streptococcus*. Sun et al. (26) replaced corn starch with 3, 6, and 9% sucrose and, after 24 h of cultivation, found that the relative abundance of *Streptococcus* had increased only in the 3% replacement group, while no significant effect was observed in the 6 and 9% replacement groups. Those results are in contrast to those of the present study; this may be due to the higher sugar content used in this experiment, which may have led to a decrease in the relative abundance of *Streptococcus*.

## Conclusions

Our study revealed that dietary sugar had a significant effect on butyric acid and gas production, while starch influenced pH and acetic acid and propionic acid levels, and the interaction of sugar and starch *in vitro* only affected DM disappearance. In terms of bacterial community composition, the relative abundance of *Treponema* and *BF311* was higher in the high-sugar group, while that of *Sphaerochaeta, Ruminobacter* was lower. The increase in butyric acid concentration may be at least partially due to an increased abundance of *Treponema*. Nonetheless, *in vitro* conditions cannot simulate the rumen absorption function, and future studies involving high sugar-based feeding trials are needed.

## Data Availability Statement

The datasets presented in this study can be found in online repositories. The names of the repository/repositories and accession number(s) can be found below: NCBI; PRJNA741061.

## Ethics Statement

The animal study was reviewed and approved by, and all experimental procedures were performed according to the Guidelines for the Care and Use of Experimental Animals of Jilin Agricultural University.

## Author Contributions

G-xQ and Y-gZ contributed to conception and design of the study. XC and X-fZ organized the database. J-nD and S-zL performed the statistical analysis. J-nD wrote the first draft of the manuscript. TW, DW, ZS, and ND wrote sections of the manuscript. All authors contributed to manuscript revision, read, and approved the submitted version.

## Conflict of Interest

XC, TW, ZS, WZ, X-fZ, and Y-gZ were employed by company Changchun Borui Science and Technology Co. Ltd. The remaining authors declare that the research was conducted in the absence of any commercial or financial relationships that could be construed as a potential conflict of interest.

## Publisher's Note

All claims expressed in this article are solely those of the authors and do not necessarily represent those of their affiliated organizations, or those of the publisher, the editors and the reviewers. Any product that may be evaluated in this article, or claim that may be made by its manufacturer, is not guaranteed or endorsed by the publisher.
