# Supplementary material for: Effects of Different Combinations of Sugar and Starch Concentrations on Ruminal Fermentation and Bacterial-Community Composition in vitro
Source: Front Nutr. 2021 Sep 3;8:727714. doi: 10.3389/fnut.2021.727714 (PMC8446659; doi:10.3389/fnut.2021.727714)
Supplement: Supplementary file 1 [file Data_Sheet_1.pdf]

Supplemental Table 1

Grouping

Sugar (g/kg) groups<br><br>Starch (g/kg)	270	250	230	210
60	St1su1	St2su1	St3su1	St4su1
80	St1su2	St2su2	St3su2	St4su2
100	St1su3	St2su3	St3su3	St4su3
120	St1su4	St2su4	St3su4	St4s u4

Supplemental Table 2

Ingredients and chemical composition of the experimental diets (% of dry matter)

Item	Groups [a]															
Ingredients (g/kg)	St1su1	St2su1	St3su1	St4su1	St1su2	St2su2	St3su2	St4su2	St1su3	St2su3	St3su3	St4su3	St1su4	St2su4	St3su4	St4su4
Leymus chinensis	457.5	474.8	492.6	510.2	439.8	457.5	475.7	493.1	421.2	439.4	457.5	475.3	402.9	421.2	438.2	457.5
Corn	205.9	213.7	221.7	229.6	197.9	205.9	214.1	221.9	189.6	197.7	205.9	213.9	181.3	189.6	197.2	205.9
Bean pulp	73.2	76.0	78.8	81.6	70.4	73.2	76.1	78.9	67.4	70.3	73.2	76.0	64.5	67.4	70.1	73.2
DDGS	114.4	118.7	123.2	127.6	109.9	114.4	118.9	123.3	105.3	109.8	114.4	118.8	100.7	105.3	109.6	114.4
Premix composition[b]	45.7	47.5	49.3	51.0	44.0	45.7	47.6	49.3	42.1	43.9	45.7	47.5	40.3	42.1	43.8	45.7
Corn starch	73.2	49.4	24.6	0.0	79.2	54.9	30.4	5.9	85.9	61.5	36.6	12.4	92.7	67.4	43.8	18.3
Sucrose	5.5	3.8	2.0	0.0	25.5	23.8	21.9	19.7	46.3	43.9	42.1	39.9	66.9	64.9	63.1	60.4
Rumen bypass fat	2.9	1.9	1.0	0.0	4.0	2.9	1.9	0.8	5.1	4.0	2.9	1.9	6.4	5.1	4.1	3.0
Corn gluten meal	21.7	14.6	7.2	0.0	29.0	21.7	13.3	6.9	36.6	29.3	21.7	14.3	44.3	36.6	29.8	22.0
Chemical composition（g/kg）																
Sugar	60.0	60.0	60.0	60.0	80.0	80.0	80.0	80.0	100.0	100.0	100.0	100.0	120.0	120.0	120.0	120.0
Starch	270.0	250.0	230.0	210.0	270.0	250.0	230.0	210.0	270.0	250.0	230.0	210.0	270.0	250.0	230.0	210.0
Non-structural carbohydrates	330.0	310.0	290.0	270.0	350.0	330.0	310.0	290.0	370.0	350.0	330.0	310.0	390.0	370.0	350.0	330.0
Crude protein	128.8	128.8	128.8	128.9	128.8	128.8	128.8	128.8	128.8	128.8	128.8	128.8	128.7	128.8	128.9	128.9
Ether extract	32.1	32.1	32.2	32.3	32.1	32.1	32.1	32.1	32.0	32.1	32.1	32.1	32.3	32.0	32.1	32.2
Acid detergent fiber	203.9	211.8	220.1	228.5	195.8	203.9	212.1	220.5	187.3	195.5	203.9	212.1	178.8	187.3	195.1	203.8
Neutral detergent fiber	340.5	353.8	367.7	381.7	326.9	340.5	354.3	368.3	312.6	326.5	340.5	354.3	298.5	312.6	325.7	340.4
ME (MJ/kg)	18.57	18.47	18.39	18.29	18.66	18.55	18.46	18.65	18.62	18.52	18.65	18.62	18.44	18.36	18.52	18.55

[a] Groups: St1=Diet of 27% starch; St2=Diet of 25% starch; St3=Diet of 23% starch; St4=Diet of 21% starch; Su1=Diet of 6% sugar; Su2=Diet of 8% sugar; Su3=Diet of 10% sugar; Su4=Diet of 12% sugar.

[b] Premix composition (per kilogram): $FeSO_4$ 8453 mg, $CuSO_4 \cdot 5H_2O$ 1480 mg, $MnSO_4$ 13,241 mg, $ZnSO_4 \cdot 5H_2O$ 8294 mg, $CoCl_2$ 16 mg, KI 30 mg, Na_2SeO_3 (1% Se) 377 mg, Vitamin A 755 IU, Vitamin D 113 IU, Vitamin E 887 IU.

Supplemental Table 3

Logarithm model fitting parameters

Item	Treatments															
	St1×su1	St2×su1	St3×su1	St4×su1	St1×su2	St2×su2	St3×su2	St4×su2	St1×su3	St2×su3	St3×su3	St4×su3	St1×su4	St2×su4	St3×su4	St4×su4
	pH															
a	-0.47	-0.82	-0.50	-0.46	-0.48	-0.41	-0.43	-0.83	-0.71	-0.65	-0.42	-0.55	-0.45	-0.40	-0.45	-0.49
b	0.18	0.07	0.15	0.17	0.17	0.22	0.21	0.05	0.07	0.08	0.22	0.09	0.18	0.24	0.16	0.12
c	6.87	6.79	6.74	6.69	6.85	6.82	6.76	6.62	6.81	6.79	6.79	6.65	6.87	6.84	6.76	6.66
R^2	0.96	0.97	0.97	0.95	0.97	0.96	1.00	0.98	0.98	0.96	0.98	0.97	0.98	0.99	0.99	0.97

Logarithmic model formula: $y = a\ln(bx + 1) + c$

Supplemental Table 4

Gompertz Model fitting parameters

Item	Treatments															
	St1×su1	St2×su1	St3×su1	St4×su1	St1×su2	St2×su2	St3×su2	St4×su2	St1×su3	St2×su3	St3×su3	St4×su3	St1×su4	St2×su4	St3×su4	St4×su4
Gas Production																
a	205.06	212.79	212.39	220.79	202.26	203.11	202.81	218.45	191.78	201.16	200.87	208.47	195.12	197.31	205.17	211.40
Xc	4.62	4.54	4.25	4.10	4.62	4.28	3.98	3.75	4.72	4.57	3.94	3.44	4.88	4.12	4.01	3.53
k	0.17	0.17	0.18	0.19	0.16	0.17	0.19	0.19	0.15	0.17	0.18	0.21	0.16	0.16	0.18	0.19
a×k/e	12.45	11.72	10.84	11.34	13.70	12.62	12.39	11.70	14.73	14.17	13.39	13.47	15.26	15.17	16.50	15.08
R^2	1.00	1.00	1.00	0.99	1.00	1.00	1.00	1.00	1.00	1.00	1.00	1.00	1.00	1.00	1.00	0.99
Dry matter digestibility																
a	50.00	43.97	42.74	45.28	38.78	33.09	67.02	43.94	56.99	41.94	39.74	74.13	45.46	58.93	39.75	38.38
Xc	6.72	7.15	5.19	2.55	-1.77	0.74	9.10	-0.74	5.74	-1.74	1.29	13.54	-0.84	0.24	-2.62	1.22
k	0.06	0.04	0.06	0.06	0.12	0.12	0.04	0.08	0.04	0.11	0.08	0.02	0.09	0.04	0.13	0.10
a×k/e	1.07	0.70	0.95	1.05	1.76	1.42	0.94	1.29	0.90	1.75	1.23	0.65	1.49	0.88	1.87	1.44
R^2	1.00	0.98	0.99	0.99	0.98	0.97	0.99	0.98	0.99	1.00	0.95	0.96	0.94	0.98	0.99	0.98
Propionic acid																
a	22.28	23.58	23.84	19.46	25.66	29.51	21.79	23.46	43.47	20.25	22.12	20.03	40.04	39.51	26.05	25.43
Xc	5.43	5.10	5.20	3.56	6.41	8.60	3.79	4.70	11.66	2.76	3.44	2.82	11.67	10.13	5.01	4.94
k	0.13	0.13	0.12	0.15	0.11	0.08	0.16	0.13	0.07	0.25	0.17	0.21	0.07	0.08	0.12	0.11
a×k/e	1.06	1.00	1.15	1.07	1.11	0.90	1.84	1.12	1.05	1.30	1.40	1.11	1.07	1.13	1.58	1.00
R^2	0.98	0.97	0.96	0.98	0.97	0.97	0.92	0.95	0.96	0.96	0.95	0.87	0.96	0.96	0.89	0.89
Butyric acid																
a	7.06	7.40	9.89	9.87	6.34	7.49	7.49	11.50	6.63	7.27	9.03	10.33	6.71	7.35	7.33	9.00
Xc	3.48	3.36	6.31	6.07	3.19	3.55	3.74	7.38	2.91	4.04	4.50	3.45	3.31	4.29	4.60	2.62

k	0.25	0.28	0.12	0.14	0.14	0.21	0.18	0.10	0.23	0.21	0.19	0.20	0.25	0.17	0.17	0.31
a×k/e	0.64	0.75	0.44	0.49	0.34	0.58	0.50	0.43	0.57	0.56	0.63	0.77	0.61	0.45	0.46	1.02
R^2	1.00	0.95	0.93	0.95	0.93	0.95	0.91	0.83	0.99	0.99	0.98	0.87	0.98	0.86	1.00	0.95

SGompertz model formula: $y = ae^{-e^{(-k(x-xc))}}$, $T_{max} = X_c$, $V_{max} = a \times k/e$.

Supplemental Table 5

Logistic model fitting parameters

Item	Treatments															
	St1×su1	St2×su1	St3×su1	St4×su1	St1×su2	St2×su2	St3×su2	St4×su2	St1×su3	St2×su3	St3×su3	St4×su3	St1×su4	St2×su4	St3×su4	St4×su4
TVFA																
A_1	17.39	17.85	17.59	19.74	17.59	15.82	18.34	18.83	15.57	19.23	18.98	19.42	16.87	12.21	15.85	20.08
A_2	70.08	73.02	77.04	62.98	76.93	97.19	75.02	69.90	122.37	68.18	78.49	67.97	122.42	418.29	90.01	79.77
X_0	6.09	5.66	6.45	4.94	6.33	11.53	5.97	5.18	13.68	4.35	5.61	4.95	13.81	146.23	5.80	5.28
P	2.62	2.16	1.87	4.55	1.96	1.29	2.11	3.34	1.15	5.10	2.50	3.00	1.35	0.75	1.57	2.06
Vmax	6.58	9.12	8.87	7.98	8.86	7.07	8.78	8.93	9.61	9.93	9.40	8.72	7.20	8.09	11.52	10.11
R^2	0.99	0.92	0.92	0.97	0.93	0.96	0.96	0.95	0.93	0.98	0.98	0.96	0.97	0.94	0.90	0.93
Acetic acid																
A_1	13.26	14.22	14.36	13.27	14.04	12.78	14.10	13.53	14.18	14.07	14.50	14.17	14.61	14.36	14.63	16.02
A_2	41.16	42.32	44.77	53.59	42.31	46.81	42.60	61.84	42.29	43.42	46.95	47.66	39.68	43.04	41.53	45.46
X_0	5.12	4.73	4.99	6.83	4.51	6.97	4.15	8.06	4.91	4.91	5.20	4.61	4.76	4.90	4.63	4.64
P	5.03	4.51	4.14	2.16	5.77	1.99	7.04	1.72	5.27	3.23	3.93	4.38	7.65	5.73	3.98	5.41
Vmax	7.13	7.03	6.69	3.99	9.31	3.16	12.33	3.69	7.82	5.32	6.54	8.38	10.24	8.64	6.16	8.87
R^2	0.99	0.94	0.95	0.94	0.95	0.93	1.00	0.92	0.92	0.96	1.00	0.94	0.99	0.99	1.00	0.96

SGompertz model formula: $y = \dfrac{A_1 - A_2}{\left(1 + \left(\dfrac{X}{X_0}\right)^P\right)} + A_2$, $V_{max} = X_0 \times \dfrac{P-1}{(P+1)^{\frac{1}{P}}}$.
